# Species Composition of Thrips (Thysanoptera: Thripidae) in Strawberry High Tunnels in Denmark

**DOI:** 10.3390/insects12030208

**Published:** 2021-03-02

**Authors:** Helene Nielsen, Lene Sigsgaard, Sverre Kobro, Nauja L. Jensen, Stine K. Jacobsen

**Affiliations:** 1 Department of Plant and Environmental Sciences, University of Copenhagen, Thorvaldsensvej 40, 1871 Frederiksberg C, Denmark; hnielsen1993@hotmail.com (H.N.); les@plen.ku.dk (L.S.); 2 Norwegian Institute of Bioeconomy, Høgskoleveien 7, 1433 Ås, Norway; s-kobro@online.no; 3 HortiAdvice A/S, Hvidkærvej 29, 5250 Odense SV, Denmark; nlj@hortiadvice.dk

**Keywords:** Thrips, species identification, fast-slide-preparation, molecular identification, distribution, *Frankliniella intonsa*, *Thrips tabaci*, strawberries

## Abstract

**Simple Summary:**

One of the main insect pests in protected strawberry production are thrips, but little is known about which species of thrips are present in the production system. In this study, we identified the thrips species of adults and larvae present in two strawberry cultivars at a commercial strawberry farm in Denmark. The most abundant species found were *Frankliniella intonsa*, followed by *Thrips tabaci.* The abundance of thrips peaked in July (temperature range 18–23 °C, mean humidity 65%, mean precipitation 5 mm). More thrips were found in the earlier flowering cultivar. In order to optimize control of thrips, a fundamental first step is knowing which species are present on the target crop.

**Abstract:**

Thrips are a major pest in protected strawberry production. Knowledge of thrips species composition could be instrumental for improved thrips management, but very little is known about which species are present in strawberries grown in high-tunnels in Denmark. Thrips (adults and larvae) were sampled in two strawberry tunnels of the cultivars Murano and Furore from May to August 2018, in the middle and in the edges of the tunnels. The most abundant thrips species found in the tunnels were *Frankliniella intonsa* and *Thrips tabaci* adults. *Frankliniella intonsa* were also the most frequently found species of the immatures sampled, followed by *T. tabaci* larvae, and other species. The number of thrips differed between the two cultivars, sampling times and location in the tunnel. *Frankliniella intonsa* was more abundant in the middle of the tunnels, while *T. tabaci* was more abundant in the edge of the tunnels adjacent to the field margins. The number of thrips peaked by the end of July. Both chemical and biological control should consider species composition and occurrence; hence, a fundamental first step for thrips management is to identify the species present on the target crop.

## 1. Introduction 

Thrips (Thysanoptera: Thripidae) are among the most important pests for a wide range of crops [1,2,3]. Damage occurs directly through feeding or indirectly by the transmission of tospoviruses [1,2,3]. Thrips can spread rapidly by flying, or by passive transport by introduced planting material or by the wind [1,4,5]. Once established in a new environment, thrips can produce several generations per year, depending on weather conditions and species [6,7]. 

Strawberries (Rosaceae, *Fragaria* × *ananassa* Duchesne) are grown on approximately 1200 ha in Denmark [8] and while most production is in the open field, an increasing proportion has since the early 2000s been produced in high tunnels [8,9,10]. Tunnel systems enable the growing season in the open field with harvest from June to early August to be extended until mid October [11,12]. In protected strawberry production in particular, thrips are a major pest [13]. Damage symptoms include petal browning, premature withering, fruit abortion, fruit bronzing and distortion [3,14,15]. Adult feeding on pollen causes injury to flowers, which wither and never develop into fruits [15]. The larval feeding causes fruit injury and leads to unmarketable produce, making larvae the most damaging stage.

There is a strong correlation between numbers of thrips per flower and fruit damage [16,17]. Economic injury levels (EILs) vary, and can depend on thrips developmental stage, (larval or adult), the cultivar and whether predators are present in the crop [17,18]. EILs may be higher in polythene tunnels due to higher temperatures, where thrips have a higher survival rate [15,19]. In Denmark, fruit losses up to 60–75% have been observed when none or limited crop protection has been implemented [20].

Common thrips species found in strawberries include: *Frankliniella occidentalis* (Pergande), *Frankliniella intonsa* (Trybom) and *Thrips tabaci* Lindeman [14,21]. All three are highly polyphagous species and occur throughout the globe including the Nordic countries [14,22,23]. *Frankliniella occidentalis* is an invasive species originating from the western half of North America [24,25]. It was first discovered in Europe in 1983, and in 1993 it was first recorded in northern Europe (including Denmark) where it had established in glasshouses [24]. *Frankliniella intonsa* is a major pest of tunnel-grown strawberries. This is in part due to its broad-spectrum insecticide resistance, high reproductive capacity and ability to colonize host plants in large numbers [15,26,27,28,29]. *Frankliniella intonsa* is believed to be native to Eurasia and shares similar distribution patterns, feeding habits and tospoviruses with *F. occidentalis* [30]. *Thrips tabaci* is believed to originate from the Eastern Mediterranean and was first introduced in Europe in 1889 [31]. It has been recorded as a damaging pest in strawberry in Europe by Lewis, 1997 [1] and by Benninson and Hough, 2015 [32]. *Frankliniella intonsa* and *T. tabaci* are known to compete with the more dominating *F. occidentalis* for resources [33,34]. In its absence, these two may be the most abundant species in the strawberry crop [35,36].

Currently, no chemical control options are available against thrips in tunnel produced strawberries in Denmark, while the use of natural enemies can be implemented [26,37,38,39]. The latter involves various predatory mites and the predatory hemipteran: *Orius* spp. [40,41,42,43]. Both chemical and biological control should be based on knowledge of pest biology, hence a fundamental first step for obtaining control of thrips is to identify the different species present on the targeted crop [44]. Very little is known about the species composition of thrips in strawberry produced in high tunnels in Denmark, so the aim of this study was to identify the species composition of thrips; adults and larvae, throughout a growing season in strawberry high tunnels on two common strawberry cultivars.

## 2. Materials and Methods

### 2.1. Sampling of Thrips in Strawberry Tunnels

Sampling of thrips for species identification was undertaken at a large commercial berry farm near Skælskør, Denmark (latitude: 11°19′52° E; altitude: 20 m). The farm was managed conventionally, and strawberries were grown on table tops in high tunnels covering 4 ha in total. Phytosanitary management included the use of fungicides, while no chemical pest control was applied. Besides chemical disease control, sachets of 500 *Amblyseius* (*Neoseiulus*) *cucumeris* were placed every 1–2 m, in the two tunnels as a biological pest control measure. This was done before flowering commenced. Naturally occurring predators such as *Orius* spp. and larvae of *Chrysoperla* spp. were also observed in the tunnels, by visual observations on-site.

The study was based on seasonal and temporal sampling of thrips adults and larvae. The sampling was repeated six times between May and August 2018 (sampling dates: 25 May, 25 June, 10 July, 23 July, 13 August and 31 August), in two neighbouring, parallel tunnels; one with the cultivar Murano and one with the cultivar Furore, a slightly later blooming cultivar. Because thrips species disperse differently in crops, six different locations in each of the two tunnels were sampled; front left, front right, back left, back right, middle 1 and middle 2. Each row was 204 m long, 35 cm wide and rows were 1.5 m apart. Each sampling location was approximately 2 m wide (two rows) and 5 m long, with 4 m between locations left and right. The front of the tunnel was the open end (facing West) adjacent to a dirt road and the farm, while the back was the open end (facing East) adjacent to tunnels running horizontally, with open cereal fields on the opposite side to them. 

Ten strawberry flowers and five berries were sampled at each sampling location on each of the six dates. Flowers were collected to include adult thrips, which feed on pollen and, therefore, most frequently are found here, while berries were collected to include larvae, which feed at this site. Berries were 2–4.5 cm in diameter and green to yellow in colour when sampled. Specimens were stored in plastic containers (155 mL, Ø: 69 mm) with 70% ethanol. Containers were labelled and brought back to the laboratory, where thrips were counted under a stereomicroscope and kept in 70% ethanol until slide mounting of the adult thrips. 

In addition and to support the results from the two tunnels, adult thrips from flowers were sampled from tunnel-grown strawberries at three other locations in Eastern Denmark: Hoerve (latitude: 11°22′26° E; altitude: 14 m; collected 6 June), Skaelskoer (latitude: 11°21′39° E; altitude; 22 m; collected 11 June) and Broby (latitude: 10°16′08° E; altitude: 26 m; collected 2 July), from either cultivar Murano or Favori. Another sampling by the advisory service was undertaken at the same farms in cultivars Favori and Flair. All thrips specimens were identified to species.

### 2.2. Fast-Slide-Preparation of Adults

Fast slide preparation was undertaken to prepare and mount each of the captured adult thrips onto microscope slides for later species identification. This method was based on that provided by Silveira and Haro [45] and further adapted to exclude the use of the toxic compound phenol. In addition, puncturing of the abdomen to expel body contents as well as massaging of the specimen was not necessary. The following mixture was used: 85% lactic acid (20 parts), glacial acid (4 parts) and distilled water (1 part). First, the specimen was placed in a Petri dish containing a bit of water. Using a paintbrush, the specimen was transferred onto a watch-glass containing a few drops of NaOH (5%), and then held over a flame from an alcohol lamp for 30 s. Next, it was transferred into a test tube containing a small amount of the mixture described above and held over the flame for approximately 1 min. The hottest part of the flame was avoided, and the liquid was not allowed to boil. This was especially important with the very light-coloured specimens, as they are more easily destroyed by this treatment. The specimen was then returned to the Petri dish to be rinsed in the water, and next transferred onto the side of a microscope slide to dry off a bit. A drop of Hoyer’s medium [46] was placed in the middle of the slide and the specimen transferred onto here using a brush. Fine needles were used to spread out the wings and legs and separate the antennae of the thrips before cover glass was added. Specimens were placed in an incubator at 60 °C for approximately six days, by which time, they were sufficiently transparent to see all the structures needed for morphological identification. 

### 2.3. Morphological Identification of Adults 

A combination of taxonomic keys was used to achieve correct species identification of adult thrips. In order to key to genus, a Thysanoptera drawing key by Mound et al. [22] was used. An online photo key was used to further identify thrips to species level [47]. In addition to the pest thrips identified, nine individuals of the genus *Aeolothrips* were identified, but these were excluded from further analysis as this is primarily predatory thrips and not regarded as a pest [47,48,49]. Another 55 specimens from the three additional locations with tunnel-grown strawberries were identified to species level. 

### 2.4. Molecular Identification of Two Species of Larvae 

Identification of larvae to species level is very difficult with morphological methods [44,50]. For this reason, a molecular approach was applied for species identification of larvae. Quantitative real-time polymerase chain reaction (qPCR) was used for the identification of the two most abundant thrips species, based on adult identification: *F. intonsa* and *T. tabaci*. Primers of the two species were tested against the 145 specimens of immatures collected from June to August. 

### 2.5. DNA Extraction 

DNA was extracted from individual larvae using the DNeasy Blood and Tissue Extraction Kit (QIAGEN©, Hilden, Germany). First, each larva was placed in a 1.5 mL microcentrifuge tube with three glass beads (1 mm) and run in a tissue lyser machine (TissueLyser II, QIAGEN©, Hilden, Germany) for approximately 2 min. Next, 200 μL of tissue lysis buffer ATL and 20 μL of proteinase K was added and the sample was vortexed. Samples were then incubated at 56 °C for an hour to allow for a complete breakdown of cells. Then 200 μL ethanol (96–100%) was added and vortexed thoroughly. The mixture was transferred into a DNeasy spin tube and centrifuged at 8000 rpm for 1 min. Flow-through and collection tube was discarded. The spin column was transferred into a new 2 mL collection tube. Next, 500 μL buffer AW1 was added to the spin column and centrifuged at 8000 rpm for 1 min. Run-through was discarded. Hereafter, 500 μL of buffer AW2 was added and centrifuged for 3 min at 14,000 rpm. Flow-through and collection tube was discarded. The spin column with the DNA was transferred to a new 1.5 mL microcentrifuge tube. DNA was eliminated by adding 100 μL buffer AE directly onto the DNeasy membrane. The tube was left to incubate at room temperature for 1 min and then centrifuged at 8000 rpm for 1 min. This last step was repeated once more to maximize DNA output. 

### 2.6. Quantitative Real-Time Polymerase Chain Reaction (qPCR) and Output

Primers of *F. intonsa* and *T. tabaci* developed by Yeh et al., [51] were used to determine if the sampled larvae were of either of these two species or of a different species. The specific primers, based on fragments of 18S rDNA and 5.8S rDNA, used for ITS1 fragment amplification were: TGCTTGAGCGGAACGAGCG (*F. intonsa* upstream primer sequence) + TCCACATAGCGGCGTGAAAG (*F. intonsa* downstream primer sequence) and TCTAAACAGAGGGAAAGGTG (*T. tabaci* upstream primer sequence) + AGTGTGCCAACAAGGCAATG (*T. tabaci* downstream primer sequence). The qPCR assay was carried out in a volume of 25 μL. The program of the qPCR machine (model: Mx3005P, Agilent Technologies©, Santa Clara, CA, USA) was set to that developed for the fluorescent dye Sybr green© (RealQ Plus2x Master Mix Green, Low ROX) (Ampliqon, Odense, Denmark) with a few modifications to fit the conditions of the selected thrips primers [51]. The first denaturation was 15 min at a temperature of 95 °C, followed by 45 cycles of 95 °C for 40 s, 50 °C for 50 s and 72 °C for 1 min. 

The output was analysed with the programme MxPro qPCR Software© version 4.10 (Stratagene, Agilent Technologies©, Santa Clara, CA, USA) as follows: cycle threshold (Ct) values under 40 were accepted as positive—DNA in the sample was detected and matched that of the primer. Ct values above 40 were classed as ‘negative’—DNA was detected in the sample, but the high values were likely a result of primers having copied themselves due to the high number of cycles. At 40 cycles, amplification efficiency slows down and reaches a plateau, while below 40 cycles amplification is at its most efficient [52]. Samples containing water were used as a control. Ct values between 35 and 40 required inspection of each sample by looking at individual amplification plots. Samples containing no Ct values or being negative for both primers, were classed as ‘other’, meaning that the given sample containing DNA was neither *F. intonsa* nor *T. tabaci*, but another species of thrips. The threshold for detected fluorescence (dRn) was set to 0.15 dRn for all runs. 

### 2.7. Statistical Analysis

**Figure 1:**
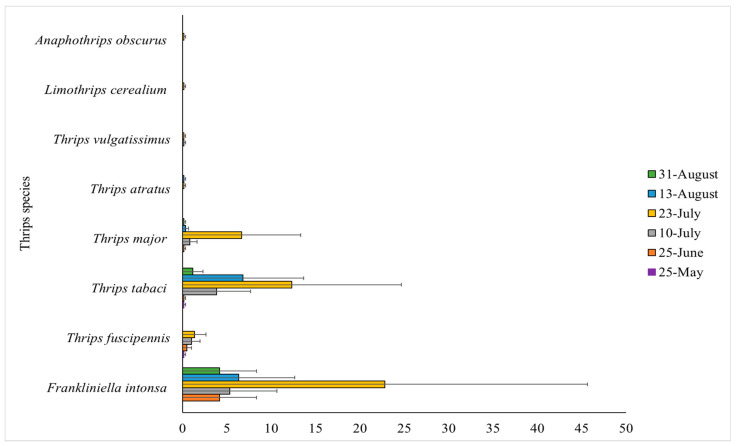
Mean number of *Frankliniella intonsa*, *Thrips fuscipennis*, *Thrips tabaci*, *Thrips major*, *Thrips atratus*, *Thrips vulgatissimus*, *Limothrips cerealium* and *Anaphothrips obscurus* in Murano and Furore according to the six sampling dates. Each bar represents the three locations in the tunnel combined (front, middle and back). Error bars represent the mean number of thrips for each species at the specific sampling date ±1 standard error of the mean.

**Figure 2:**
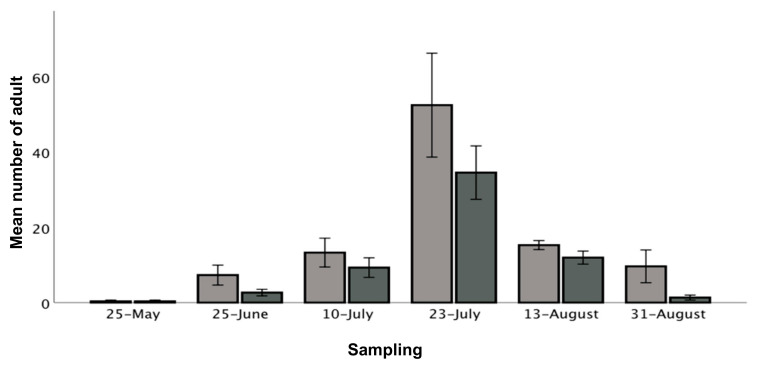
Mean number of thrips in relation to sampling time in cultivar Murano (light grey) and cultivar Furore (dark grey), May to August 2018. Error bars represent the mean number of thrips for each species at the specific sampling date ±1 SE standard error of the mean.

A generalized linear model (GLM) was applied, as data were not normally distributed, to determine if there was a significant difference in the number of thrips in relation to sampling time: 25 May, 25 June, 10 July, 23 July, 13 August and 31 August 2018; location in tunnel: front, middle and back; and the two cultivars: Murano and Furore. A negative-binominal distribution was applied for all variables and a 3-way analysis was carried out. A pairwise comparison was undertaken of mean thrips numbers according to sampling time and location in the tunnels. Since the two tunnels had different cultivars, no distinction could be made between tunnel and cultivar. Furthermore, as counts in the same locations were repeated over time, a repeated measures analysis was undertaken, and statistical outputs are thus based on this. GLM was also applied to determine if there was a significant difference between the numbers of *F. intonsa* and *T. tabaci* with the two fixed variables sampling time and location, as described above. Tunnel was included as a random effect. A pairwise comparison of numbers of *F. intonsa* and *T. tabaci* according to sampling time and location in tunnel was also carried out. Error bars in Figure 1 and Figure 2 refer to each mean plus and minus one standard error of the mean. The model output had a degrees of freedom of one. P-values and z-scores are reported in the results.

Models were validated by goodness of fit tests and omnibus tests. Pearson chi square showed that the models were slightly over-dispersed (1.396), but, as this was marginal (below 1.5), the models were accepted. Overall, the omnibus tests showed highly statistically significant models. 

A one-way analysis of variance (ANOVA) was used, as data was normally distributed, to determine if the numbers of *F. intonsa, T. tabaci* and ‘other’ larvae respectively, differed according to the three sampling months: June, July and August. A Tukey post hoc test was used to see how the number of *F. intonsa, T. tabaci* and ‘other’ larva species differed according to each of the sampling months. All analyses were carried out in IBM© SPSS© Statistics version 25 (Armonk, NY, USA).

## 3. Results 

### 3.1. Species Composition of Thrips Adults and Larvae during the Production Season

Eight different species were morphologically identified from the 482 adult thrips sampled. These included both males and females, with the majority being females. The most abundant species in both tunnels were *F. intonsa* (53.3% of the total number of adult thrips) and *T. tabaci* (31.5% of the total number of adult thrips). *Thrips fuscipennis* Haliday was also recorded and made up 3.7% of the total number of adults thrips. Only a few individuals of the species *Thrips atratus* Haliday (0.4% of the total number of adult thrips), *Thrips vulgatissimus* Haliday (0.4% of the total number of adult thrips), *Limothrips cerealium* Haliday (0.2% of the total number of adult thrips) and *Anophothrips obscurus* (Mueller) (0.2% of the total number of adult thrips) were found (Figure 1). *Frankliniella intonsa* occurred in higher numbers earlier than other species (Figure 1). The highest density of *F. intonsa* and *T. tabaci* was in July (*p* < 0.001, z-scores = 7.42, 5.96 respectively). *Thrips tabaci* densities were higher in the middle of August compared to the end of August (*p* < 0.001, z-score = 4.32), while the same was not true for *F. intonsa* densities (*p* = 0.106, z-score = 1.62). 

The tunnel with Murano had more *F. intonsa* than tunnel 2 (*p* < 0.001, z-score = 5.35), while numbers of *T. tabaci* were similar in the two tunnels (*p* = 0.365, z-score = 0.91). Furthermore, a higher number of *F. intonsa* was found in the front (14.2 ± 5.0 SE) and middle (21.7 ± 11.0 SE) of the tunnels compared to the back (7.0 ± 3.9 SE) (*p* < 0.001, z-scores = 3.55, 5.78 respectively). In contrast, fewer *T. tabaci* were found in the front (8.0 ± 3.7 SE) and middle (6.7 ± 3.4 SE) compared to the back (10.7 ± 5.5 SE) (*p* = 0.044, 0.020; z-scores = −2.02, −2.33, respectively).

A total of 145 larvae were collected in the two tunnels. The number of ‘positive ‘samples (matched one of the primers) was 112, the number of ‘negative’ samples (did not match any of the primers) was 24, and samples needing revision (Ct values were between 35 and 40) were 19. The revision was undertaken by inspection of individual amplification plots. Based on the molecular identification, the larvae were divided into three categories: *F. intonsa*, *T. tabaci* and ‘other’, the latter consisting of all other species of larvae. The majority of larvae sampled were identified as *F. intonsa*, while *T. tabaci* and ‘other’ occurred to a similar extent (Table 1). The numbers of *F. intonsa* and *T. tabaci* larvae, each differed according to the three sampling months (June, July and August) (*p* < 0.001). No larvae were found in May and no *T. tabaci* larvae were found in June. The number of ‘other’ larva species did not differ according to sampling time (*p* = 0.857) (Table 1). 

### 3.2. Species Found at Other Locations in Denmark

From the first location of the three additional sites, 29 *F. intonsa* and 2 *T. tabaci* were identified from the cultivar Murano. Ten *F. intonsa,* 5 *T. tabaci,* and 2 *T. fuscipennis* were identified from the second location from the cultivar Murano, and from the third location, Broby, 5 *F. intonsa* and 1 *T. fuscipennis* were identified from Favori. At all three locations, *F. intonsa* was found to be the most abundant. In addition, a thrips sampling at the end of June found 4 *F. intonsa,* 2 *T. fuscipennis*, and 1 *T. major* (Skaelskoer) from Favori, and 8 *F. intonsa* and 1 *T. fuscipennis* (Broby) from Favori. From the third location (Hoerve), 4 *F. intonsa*, 5 *T. fuscipennis* and 10 *F. occidentalis* was found in the cultivar Flair, indicating that the choice of cultivar has an impact on the species composition of thrips, while this remains to be investigated. 

### 3.3. Effect of Cultivar, Time and Location on the Overall Density of Adult Thrips

Total thrips density, which may be defined as the total number of adult thrips in 10 flowers at each of the six sampling sites at each of the six sampling dates, peaked by the end of July in both tunnels (*p* < 0.001, z-score = 8.14). Murano had a higher number of thrips than Furore (16.4 ± 4.6 and 10.1 ± 3.1 respectively) (*p* < 0.001, z-score = 3.49) (Figure 2). Pairwise comparisons of densities show that there were more thrips on 10 July, 23 July and 13 August compared to the end of the season, 31 August (*p* = 0.003, *p* < 0.001, *p* < 0.001; z-scores = 2.96, 8.14, 3.62 respectively). There were fewer thrips on 25 May than 31 August (*p* = 0.000, z-score = −3.63), while there was no difference in the number of thrips on 25 June and 31 August (*p* = 0.854, z-score = −0.18) (Figure 2). Thrips density in the middle of tunnels was significantly higher than the back of the tunnels (means ± SE per tunnel (average for May–August): front = 12.3 ± 4.7 thrips, middle = 17.1 ± 9.2 thrips, back = 10.3 ± 5.5 thrips) (*p* = 0.010, z-score = 2.56). Thrips densities in the front and back of tunnels were not significantly different (*p* = 0.111, z-score = 1.59). 

## 4. Discussion

### 4.1. Species Composition and Distribution of Thrips

*Frankliniella intonsa* was the most abundant thrips species found in the strawberry tunnels, both in terms of adults (53.3% of all adults sampled) and larvae (60% of all larvae sampled) (Figure 1; Table 1). The identification of adult thrips sampled from tunnel-grown strawberries at three other strawberry farms supported these findings. Globally, only few studies have reported findings of *F. intonsa* in strawberry, and even fewer in high tunnel systems, of which only one in Scandinavia, which also reported *F. intonsa* to be a dominating species [53]. A high number of *F. intonsa* in greenhouse strawberry was recorded by Lim and Mainali [54] in Korea, in outdoor commercial fields by Mintu and Reyes [55] in the Philippines and by Atakan [56] in the Eastern Mediterranean area of Turkey. Only Atakan [56] also found *F. occidentalis*, but in low numbers. The highest injury to strawberry already occurs approximately two weeks after flowering [15]. Berries are green at this time and feeding by larvae causes a limited shelf life at maturity. Here, larvae of *F. intonsa* occurred earlier and were more abundant than the other species found and are likely to have inflicted early damage to green berries in the tunnels and may thus be responsible for the highest injury levels to the crop. 

The second most sampled adult species was *T. tabaci* (31.5% of all adults sampled) (Figure 1), which also reproduced in the tunnels, with larvae found in equal numbers to “other species” (20% of all larvae sampled) (Table 1). This suggest that this species is also an established species in the strawberry tunnels. It is important to identify larvae to species level, as their presence reveals whether adults are reproducing in the tunnels, and thus become an established species. An improved method for fast slide preparation was created in this study. The method excluded the use of phenol and was overall faster due to the exclusion of abdomen puncturing and the intermediate steps of specimen massaging to evaluate transparency. It is important to state, however, that slides obtained using this method are only temporary and not permanent [45,57]. The oldest insect-mounted slides preserved by this method were 34 years old [58]. 

The two species *F. intonsa* and *T. tabaci* co-occur in different crops, but in most cases *F. intonsa* is the most frequently found species of the two [53,56,59,60,61]. The third and fourth most common adult species found were *T. fuscipennis* and *T. major*, two species of thrips that are also quite common pests of strawberry [14,15,62] (Figure 1). These species (and perhaps others) likely shared the 20% “other species” of the total percentage of sampled larvae reported in Table 1. 

Additional species sampled included *T. atratus* and *T. vulgatissimus* (Figure 1), both commonly reported in tunnel-grown strawberry [21,53,63,64]. Finally, few individuals of *L. cerealium* and *A. obscurus* were sampled. These species are generally found on cereals and grasses and could have flown in from nearby fields [14]. They are mostly reported as only occasional visitors in strawberries [47,62]. *Frankliniella occidentalis* are commonly reported as a key pest species of thrips in strawberries [19]. In the present study, no *F. occidentalis* were found, likely because it is difficult for *F. occidentalis* to establish permanent field populations in Northern Europe, including Denmark [6,26,65]. In the UK, a recent study suggests that *F. occidentalis* is able to survive the winter on weeds in polythene tunnels, and this may enable them to build up large populations in second-year everbearing strawberry [19]. Such an occurrence may potentially spread to Denmark if winters become milder. Competition may also explain the absence of *F. occidentalis*, as *F. intonsa* has been reported to outcompete *F. occidentalis* [30,36], while *T. tabaci* have been shown to have lower survival rates in the presence of *F. occidentalis* due to competition [34,35].

Although few *F. occidentalis* were sampled as part of the additional collections (Hoerve), this species was absent from three other cultivars (Murano, Furore and Favori). Flair is a cultivar that may overwinter [66]. A mild winter could have caused *F. occidentalis* to survive the winter and build up its population that year.

### 4.2. Difference in Abundance of Frankliniella Intonsa and Thrips Tabaci 

A combination of morphological and molecular tools allowed us to identify *F. intonsa* followed by *T. tabaci* as the two most common species in strawberry high tunnels. *Thrips tabaci* was more abundant in different areas than *F. intonsa* (back vs. front and middle, respectively). Interspecific competition between the two species may explain the spatial differences observed. *Frankliniella intonsa* has been reported to outcompete *F. occidentalis* in different ways, including higher food intake, higher reproductive activity and fecundity, as well as greater survival under fluctuating temperatures [30,36]. In addition, this species has been found to display pollen guarding when interacting with *F. occidentalis* on the same area of a host plant [36]. In contrast to *F. intonsa, T. tabaci* has been observed to have lower survival rates when interacting with *F. occidentalis* [34,35]. Insects are known to stay away from areas where a superior competitor is present [67,68]. One may speculate that *T. tabaci* may thus actively avoid areas with abundant *F. intonsa*. 

### 4.3. Temporal and Spatial Patterns of Adult Thrips

The July peak of infestation is in accordance with other studies in temperate climates [19,53,63,69]. It is likely that remaining flowers in tunnels in mid-August maintained thrips numbers above those in May. The sharp decline in thrips numbers by the end of August was likely due to an observed decline in the number of flowers and larvae dropping to the ground to commence pupation [19,53,63,69]. The earlier flowering Murano had more thrips than Furore. Sampson and Kirk [69] observed that newly opened flowers and flowers standing tall are preferred by thrips due to fresh pollen and higher visibility, respectively. The cultivar Murano is in particular known to stand tall, resulting in highly visible flowers [70]. Factors such as leaf quality and plant volatiles may also impact thrips occurrence [71,72]. The highest thrips densities were found in the middle of tunnels. Similar observations have been made of the diamondback moth and the cabbage webworm in different crops [73,74]. The slope at the study site may have caused the middle of the tunnels to be warmer than the area by the tunnel openings due to the upwards wind, a factor which possibly also contributed to a higher number of thrips in the centre of the tunnels. Based on the results presented here, we found no indication of thrips entering the tunnels from outside, where an early aggregation may be expected in field edges (tunnel openings) unlike what was found in the present study. Therefore, we recommend that further investigations include studies of cultivar and spatial distribution, as well as an expansion of the number of sampling years.

## 5. Conclusions

This study provides new knowledge about the species composition of thrips in strawberry high tunnels in Denmark. Correct larval species identification is important, as their presence reveals whether adults are reproducing in the tunnels, and thus become an established population. Knowledge of species behaviour and interactions, and most importantly selection of efficient biocontrol agents against these thrips species, remains to be investigated in order to optimize pest control strategies. 

## Figures and Tables

**Table 1:** Counts of larvae of the species: *Frankliniella intonsa*, *Thrips tabaci* and ‘Other’, including percentage of these from June until August, and p-values between months of each species. Total count per observation was 145 larvae in the two tunnels collectively. No larvae were found in May.

Species	June	July	August	Total	Percentage of Larvae	*p*-Values
*Frankliniella intonsa*	36	34	17	87	60%	June and July *p* > 0.05
						June and August *p* < 0.001
						August and July *p* < 0.001
*Thrips tabaci*	0	8	20	28	20%	June and July *p* > 0.05
						June and August *p* < 0.001
						August and July *p* < 0.001
Other	8	11	11	30	20%	No difference between months (*p* > 0.05)

## Data Availability

Data will be made available in a digital data repository upon acceptance of the manuscript.
